# Development of a Surface Plasmon Resonance Biosensing Approach for the Rapid Detection of Porcine Circovirus Type2 in Sample Solutions

**DOI:** 10.1371/journal.pone.0111292

**Published:** 2014-10-29

**Authors:** Jiandong Hu, Tingting Wang, Shun Wang, Mingwen Chen, Manping Wang, Linying Mu, Hongyin Chen, Xinran Hu, Hao Liang, Juanhua Zhu, Min Jiang

**Affiliations:** 1 Department of Electrical Engineering, Henan Agricultural University, Zhengzhou, China; 2 State key laboratory of wheat and maize crop science, Zhengzhou, China; 3 Zhengzhou Oriole Electronic (Group) Joint-Stock Co., Ltd, Zhengzhou, China; 4 College of Animal Husbandry and Veterinary, Henan Agricultural University, Zhengzhou, China; 5 School of Human Nutrition and Dietetics, McGill University, Ste Anne de Bellevue, Quebec, Canada; 6 Department of Electronic and Telecommunications, University of Gavle, Gävle, Sweden; 7 College of life sciences, Henan Agricultural University, Zhengzhou, China; CNR, Italy

## Abstract

A sensitive and label-free analytical approach for the detection of porcine circovirus type 2 (PCV2) instead of PCV2 antibody in serum sample was systematically investigated in this research based on surface plasmon resonance (SPR) with an establishment of special molecular identification membrane. The experimental device for constructing the biosensing analyzer is composed of an integrated biosensor, a home-made microfluidic module, and an electrical control circuit incorporated with a photoelectric converter. In order to detect the PCV2 using the surface plasmon resonance immunoassay, the mercaptopropionic acid has been used to bind the Au film in advance through the known form of the strong S-Au covalent bonds formed by the chemical radical of the mercaptopropionic acid and the Au film. PCV2 antibodies were bonded with the mercaptopropionic acid by covalent -CO-NH- amide bonding. For the purpose of evaluating the performance of this approach, the known concentrations of PCV2 Cap protein of 10 µg/mL, 7.5 µg/mL, 5 µg/mL, 2.5 µg/mL, 1 µg/mL, and 0.5 µg/mL were prepared by diluting with PBS successively and then the delta response units (ΔRUs) were measured individually. Using the data collected from the linear CCD array, the ΔRUs gave a linear response over a wide concentration range of standard known concentrations of PCV2 Cap protein with the R-Squared value of 0.99625. The theoretical limit of detection was calculated to be 0.04 µg/mL for the surface plasmon resonance biosensing approach. Correspondingly, the recovery rate ranged from 81.0% to 89.3% was obtained. In contrast to the PCV2 detection kits, this surface plasmon resonance biosensing system was validated through linearity, precision and recovery, which demonstrated that the surface plasmon resonance immunoassay is reliable and robust. It was concluded that the detection method which is associated with biomembrane properties is expected to contribute much to determine the PCV2 in sample solutions instead of PCV2 antibody in serum samples quantitatively.

## Introduction

Porcine circovirus (PCV) can be categorized into two genotypes: porcine circovirus type 1 (no pathogenicity) and porcine circovirus type 2 (pathogenicity) according to the pathogenicity, antigenicity and nucleotide sequence. Porcine circovirus type 2 (PCV2) infection has been reported in many countries with a significant commercial production industry, which leads to high production losses for producers [1], [2]. Moreover, this PCV2 was closely related to post weaning multisystemic wasting syndrome (PMWS) disease which generally occurred worldwide [3], [4]. The main characteristics of the disease (PMWS) were progressive emaciation and multiple system injury, having caused serious economic losses to the swine industry [5]. Therefore, it is of great significance to establish a rapid method for the detection of PCV2, providing the basis for prevention of the disease.

A variety of methods have been used intensively to detect the PCV2 [6]–[9]. An immunoperoxidase monolayer assay (IPMA) based diagnostic kit for the detection of PCV2 antibody has been developed under optimized conditions. The IPMA kit offers a convenient tool for the survey of PCV2 epidemiology and the evaluation of vaccines. After that, a monoclonal antibody (Mab)-based blocking enzyme-linked immuno sorbent assay (ELISA) was performed for the detection of serum neutralizing antibodies of PCV2, where the Mab with neutralizing activity was used as the target antibody to obtain the sensitivity and specificity of 98.8% and 88.5%, respectively. A significant positive correlation was found between results of the blocking ELISA and the serum neutralization assay [10], [11]. A fast, sensitive and indirect ELISA method for the detection of the specificity, repeatability and stability of the PCV2 antibody of pigs has been established systematically. The colloidal gold method was used to detect the PCV2, including preparing gold labeled beads by spraying colloidal gold on the glass fiber for staphylococcal protein A (SpA), a type I membrane protein from the bacterium staphylococcus aureus, and producing colloidal gold immunochromatographic test strip for the detection of PCV2 antibodies has been constructed [12], [13].

At present, PCR is widely used for the detection of PCV2 in serum samples. A real-time polymerase chain reaction (PCR) method for the quantification of PCV2 has introduced, to distinguish the PCV1 and PCV2 in the serum sample, which would significantly speed up the process of clinical diagnosis [14]. Light Upon eXtension real-time PCR (LUX real-time PCR) assay was developed for the detection of PCV2 [15], [16]. LUX real-time PCR, similar to the TaqMan PCR, was more specific for generating the fluorogenic signal than SYBR Green PCR. A novel multiple real-time PCR system based on SYBR Green I that allows the simultaneous detection 5 viruses including PCV2, porcine parvovirus, pseudorabies virus and Torque teno sus virus 1 and 2 was validated with the limit of detection ranged from 3.65×10^3^ to 5.04×10^3^ copies of DNA template per reaction [17], [18]. A method of loop-mediated isothermal amplification (LAMP) was employed to develop a rapid and simple detection system for porcine circovirus type 2 (PCV2) with a higher sensitivity. No cross-reactivity was observed from the samples of other related viruses including porcine circovirus type 1 (PCV1), porcine parvovirus (PPV), porcine pseudorabies virus (PRV) and porcine reproductive and respiratory syndrome virus (PRRSV) [19]. The optical methods for the detection of viruses were increasingly aroused researcher's interests. A surface plasmon resonance imaging (SPRI) assay was developed for measuring porcine circovirus type 2 (PCV2) antibody in serum sample using a recombinant capsid protein as an antigen with the correlation coefficient of 0.911 [20].

This paper presented a non-destructive, label free, and real-time optical surface plasmon resonance (SPR) immunoassay for detecting the PCV2 with the biological membrane adopted predominantly a self-assembly, which provides a breakthrough method for detecting viruses in PCV2-contained sample solutions, such as the PCV2 separated from the animal lymph nodes, manure or blenna narium.

## Materials and Methods

### Materials

2 mg/mL PCV2 Cap protein (the only structural protein of porcine circovirus 2) and 1 mg/mL PCV2 antibody were purchased from TaKaRa Biotechnology (Dalian) Co., Ltd. The PCV2-conatined samples at the different concentrations of 10 µg/mL, 7.5 µg/mL, 5 µg/mL, 2.5 µg/mL, 1 µg/mL, and 0.5 µg/mL are prepared by diluting PCV2 Cap protein with PBS. The PCV2 was isolated from the manure, lymph nodes or blenna narium of PCV2-infected pigs, and identified in the Key Laboratory for Animal-derived Food Safety of Henan Province affiliated to College of Animal Husbandry and Veterinary, Henan Agricultural University, China. The 0.4 mol/L mercaptopropionic acid (MPA), 1 mol/L ethanol amine(Eth, pH8.5), 0.4 mol/L 1-ethyl-(3-3-dimethylaminopro-pyl) carbodiimide hydrochloride (EDC), 0.1 mol/L N-hydroxysuccinimide (NHS), 0.01 mol/L phosphate buffered saline (PBS) buffer(pH7.4), 0.1 mol/L NaOH solution(pH13), sodium dodecyl sulfonate SDS solution(4%), H_2_SO_4_, and H_2_O_2_ were purchased from Shanghai General Chemical Reagent Factory (Shanghai, China).

### Methods

All procedures were approved by the Henan Agricultural University Animal Care and Use Committee.

PCV2-containing samples were collected by the Key Laboratory for Animal-derived Food Safety of Henan Province affiliated to College of Animal Husbandry and Veterinary, Henan Agricultural University, China and transferred to us. The samples were collected as part of standard veterinary care, complying with the standard procedures issued by the Henan Agricultural University Animal Care and Use Committee.

**Figure 1:**
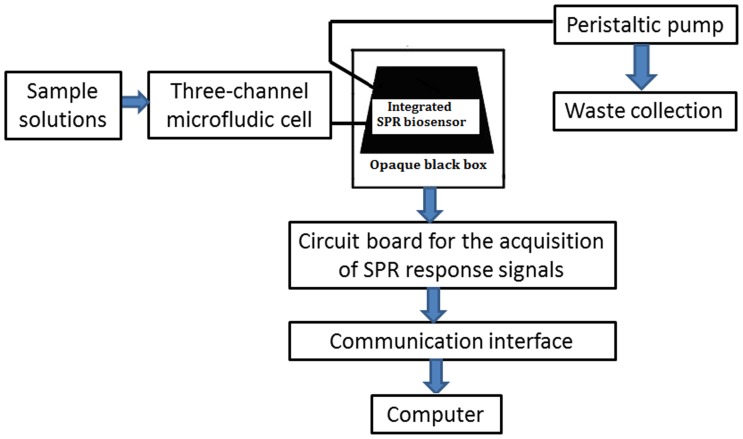
Schematic flow chart of the biosensing system designed by using modularized unit.

SPR is a kind of physical optics phenomenon, when the incident light at the critical angle is incident on an interface between two different medias (such as metal silver deposited on glass surface or gold film), which can cause resonance among metal free electrons, and the energy of reflected light will be greatly reduced in a certain range angle. SPR signal changes with refractive index on the metal surface, in addition, the change of refractive index is proportional to biological molecular mass binding on the metal surface, thus specific signal of biomolecular interaction can be obtained through dynamic changes of biological reaction process of SPR angle. In order to design one low cost and compact setup based on surface plasmon resonance immunoassay to detect PCV2 efficiently, the novel modularized biosensing system was developed in this experiment. A new high integrated circuit board with USB interface was developed to connect the SPR biosensor. This biosensing system was mainly composed of a SPR biosensor, a three-channel microfluidic cell, and a circuit board for the acquisition of SPR response signals. The modularization of this biosensing system has been realized to improve the measurement reproducibility. The shift change relationship between the resonant dip and the refractive index changed over the biosensor surface, which is caused by the binding of analyte to a receptor immobilized on the biosensor surface, is strongly proportional. The modularized unit is easily obtained by packaging with dark plastic board, which is indicated using the dash line (See Figure 1). The working temperature for this biosensor is controlled to be 25±0.5°C to keep the biosensor working effectively [21]. The home-made three-channel microfluidic cell (2.5 µL) was embedded in this modularized unit. The data from the linear CCD were sent out via the USB interface to the computer in reference to the published paper [21], [22].

#### Preparation of the biosensing membrane

**Figure 2:**
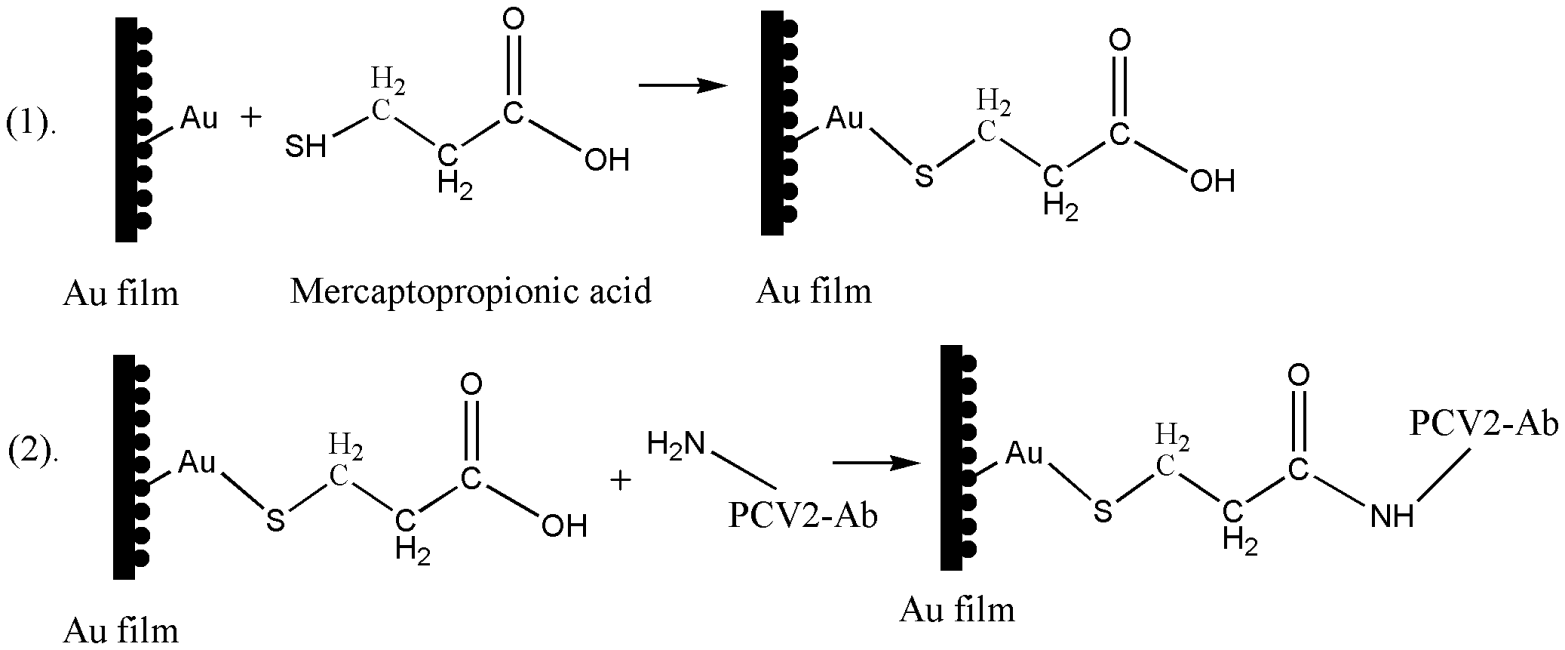
The biomembrane structure for the preparation of the biosensing membrane for the detection of the PCV2. (1) Au-S bond was formed after the association occurred between the thiol of mercapto propionic acid with the Au atom, where the 1 mol/L mercaptopropionic acid was used. (2) PCV2 antibodies were immobilized on the gold film surface by binding the peptide bond.

The procedures for preparing the biosensing membrane are described as follows: (1) dropwise add the solution of H_2_SO_4_:H_2_O_2_ (volume ratio 7∶3) on the gold film surface of the biosensor chip, aside 0.5 h before being used. Then rinse the gold film clean with deionized water and drying it with N_2;_ (2) insert the clean biosensor into the clamp which was specially designed for holding the biosensor compactly, inject PBS buffer over the biosensor surface for about 5 min until the baseline of response signals was obtained stably at the flowing speed of 30 *µ*l/min. The 1 mol/L MPA was injected successively after the response signals monitored by the computer were approximately constant for 0.5 h; (3) inject 0.4 mol/L EDC:0.1 mol/L NHS(volume ratio1∶1) to active the carboxyl group for 1 h until the baseline remaining unchanged instead of the PBS buffer; (4) inject the PBS buffer to wash down the residual EDC:NHS until the response signals unchanged again, then the 10 mg/mL PCV2 antibody was injected through over the biosensor surface, and keep the PCV2 antibody in staying on the biosensor surface for 2 h; (5) inject the PBS to wash down the unbound antibodies in the solution, then the Eth was injected to seal off the carboxyl for 20 min. After conducting the procedures step by step, the functionality of the biomembrane for the detection of PCV2 is accomplished (See Figure 2). The lifetime of the biosensing membrane will depend strongly on the Au film of this biosensor, including the thickness and homogeneousness of the Au film. In this experiment, the 0.1 mol/L NaOH solution was used to regenerate the biosensing membrane.

## Results and Analysis

### Establishment of the standard curve

By taking PCV2 Cap protein as the standard detected sample, a layer of PCV2 antibody was immobilized on the biosensor surface which was deposited of 50 nm gold film as the specific acceptor for identifying the PCV2 Cap protein. The standard solutions at different PCV2 concentrations of 10 µg/mL, 7.5 µg/mL, 5 µg/mL, 2.5 µg/mL, 1 µg/mL, and 0.5 µg/mL were obtained respectively by diluting PCV2 Cap protein at the concentration of 2 mg/mL. Firstly the PBS buffer was flowed successively over the biosensor surface to obtain the baseline signals. Then the standard solutions were flowed through the Au film surface of the biosensor by peristaltic pump, successively to obtain the changes of response units (RU) resulted from the changes of the refractive index which was changed by the different concentrations of analytical solutions on the surface of the Au film. RU was computed based on the following formula.
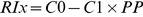
(1)where RIx is the refractive index of an unknown sample, C0 = 1.36476912, C1 = 4.37627713×10^4^ are the constants determined by the SPR configuration, and PP is the pixel position number obtained from the linear CCD sensor.
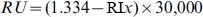
(2)where 1.334 was the refractive index of deionized water [21].

**Figure 3:**
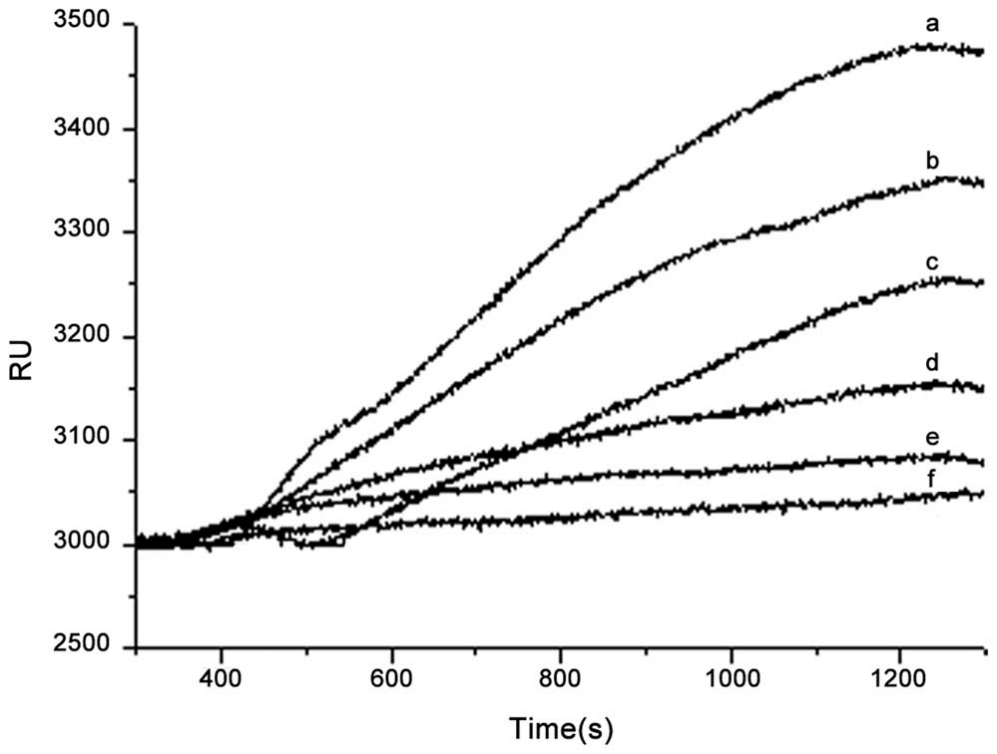
Sensor response diagram obtained from different concentrations of PCV2 Cap proteins. a∼g represent the sensorgram obtained from concentrations of 10.0 µg/mL, 7.5 µg/mL, 5.0 µg/mL, 2.5 µg/mL, 1.0 µg/mL, 0.5 µg/mL of PCV2 Cap protein, respectively.

The RUs obtained from the different known concentrations were shown in Figure 3. In this experiment, the PCV2 antibody started to associate with the PCV2 antigen, so called the detected analytes after 400 s for reaction in solutions. It was approximate to 1200 s for reaching up to the dynamic equilibrium, which indicates the end of the association process. From Figure 3, it indicated that the association curve was gradually dwindled obviously with decreasing of the sample concentration. There are distinctly differences occurring between two curves obtained from two adjacent sample concentrations, which demonstrated that a higher sensitivity can be achieved. Moreover, multisamples were also able to be continuously detected using the same biosensing membrane. The time for the measurement of one sample was around 20 min. In this experiment, the dynamic equilibrium is obtained successfully if the change of response unit (RU) in quantity is less than 5 RUs within 2 min. The response time for reaching up to the dynamic equilibrium will be reduced if the sample pipe is shortened in practical applications.

**Figure 4:**
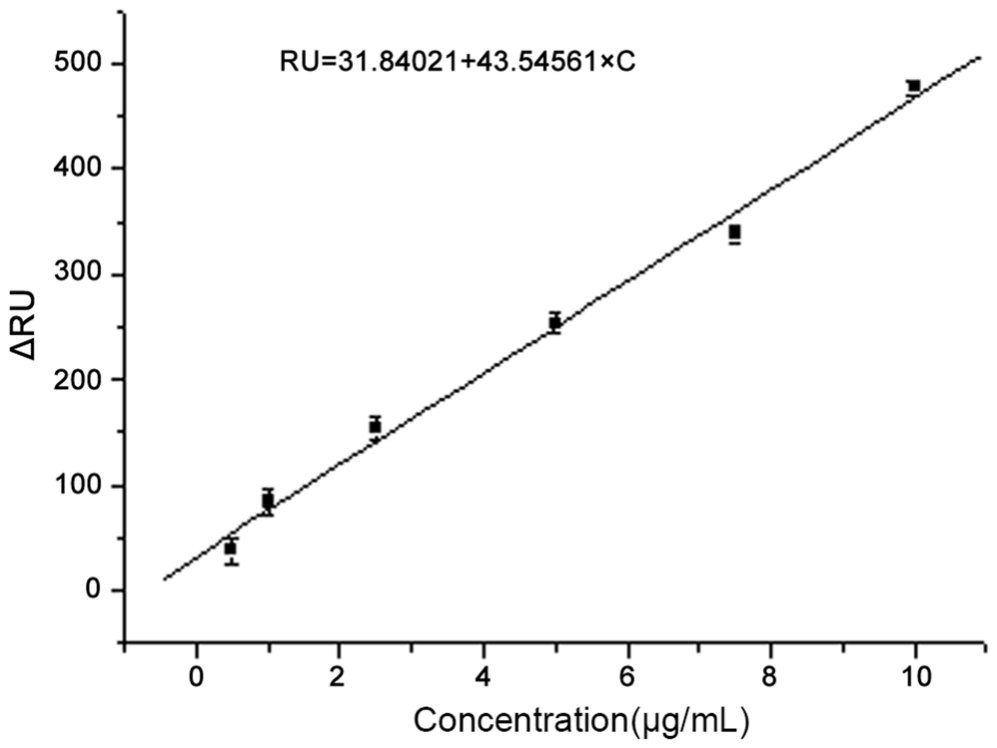
The fitting curve established by delta response units with different standard PCV2 Cap concentrations ranged from 0.5 µg/mL to 10 µg/mL.

These standard samples with concentrations of 10 µg/mL, 7.5 µg/mL, 5 µg/mL, 2.5 µg/mL, 1 µg/mL, and 0.5 µg/mL were measured repeatedly five times, respectively. The mean ΔRUs of these known concentration solutions were calculated to be 477, 338, 254, 154, 84, and 38. ΔRU refers to the sensor response induced by biomolecular binding, changing the local reflective index (RI) at the sensor interface. Importantly, a response (background response) will also be generated if there is a difference in the refractive indices of the running and sample buffers. This background response must be subtracted from the sensorgram to obtain the actual binding response. The coefficient of variation of the repeated measurement was also calculated to be 5.72%. The fitting equation ΔRU = 31.84021+43.54561×C was indicated in the Figure 4 with the R-Square of 0.99625, where C is the concentration of the sample (See Figure 4).

### Comparative analysis between SPR immunoassay and the PCV2 detection kits

In order to verify the sensitivity and specificity, the SPR immunoassay for the detection of PCV2 with self-assembled molecular recognition membrane, the known concentrations of 0.5 µg/mL, 1.0 µg/mL, 2.5 µg/mL, 5.0 µg/mL, 7.5 µg/mL, 10.0 µg/mL, and 20.0 µg/mL of PCV2 Cap protein were measured by using the SPR immunoassay and PCV2 detection kit, respectively. The results were shown in Table 1.

**Table 1:** Contrast resolution obtained from both SPR immunoassay and PCV2 detection kits.

Concentration/µg/mL	20.0	10.0	7.5	5.0	2.5	1.0	0.5	0.1
PCV2 detection Kits	+	+	+	−	−	−	−	−
SPR immunoassay	+	+	+	+	+	+	+	+ −

From the Table 1, the detection results obtained from the concentration of 5 µg/mL PCV2 cap protein was negative by using PCV2 detection kits. However, the sample with the known concentration of 0.5 µg/mL PCV2 cap protein was positive and uncertainty occurred at the concentration of 0.1 µg/mL. It showed that the SPR immunoassay can be used to detect the sample with the low concentration compared with the PCV2 detection kits. The theoretical limit of detection was calculated to be 0.04 µg/mL for the SPR immunoassay (LOD = 3σ/b, σ represents the signal noise and b represents the sensitivity) [22].

### Recovery experiments

A recovery test is a bioassay for determining the efficiency of a biosensor system in detecting a specific analyte. The recovery of the analyte is the difference between the measured concentration and actual concentration after a known dose of the analyte is added to a biological matrix. In this experiment, for evaluating the feasibility of this SPR immunoassay in actual applications, the following experiments designed for calculating the recovery rate were arranged. Firstly, the prepared 100 fold dilution of sample without PCV2 Cap protein was measured repeatedly five times and the ΔRUs were 82, 85, 75, 72, and 81, respectively. Taking a sample with 1.0 µg·mL^−1^ PCV2 Cap protein to measure repeatedly 5 times following the above procedures ΔRUs were 151, 153, 147, 147, 154 were obtained, respectively.

Therefore, the calculated recovery rates ranged from 81.0% to 89.3% were calculated from the formula with reference to the paper published by Jiandong Hu [22]. It was validated that the SPR immunoassay is feasible to detect the PCV2 in sample solutions rather than the PCV2 antibody in serum samples efficiently.

## Conclusions

The surface plasmon resonance immunoassay for the detection of porcine PCV2 in piglets has been systematically investigated in this paper. This study adopts immunological technique to implement chemical treatment on the biosensor by modifying the PCV2 antibody on a layer of gold film, which establishes the method for detection PCV2 with non-destructive, label-free, and real-time monitoring. To obtain the response sample values through monitoring binding response signal changes between PCV2 antibody and PCV2 Cap protein, the RU is substituted into the established standard concentration curve to obtain the concentration which needs to be detected. In conclusion, this is the first detailed investigation of biomembrane structure for the detection of PCV2 in sample solutions, which was contained in organic tissue. A linear relationship between the concentration of Cap protein and the RUs from the PCV2-contained samples was established, the correlation coefficient is 0.99625 and the theoretical limit of detection is 0.04 µg/mL, which is far below than the PCV2 kit method. In addition, the recovery rate range of 81%–89.3% has obtained, which meets the requirement of the actual sample detection. In comparison with the traditional molecular biology methods, SPR biosensor technology has the characteristics with label-free, nondestructive, rapid, on-line detection etc, which provides a novel method for detecting PCV2 directly in sample solution instead of a PCV2 antibody in serum sample.

## References

[1] Pedro MP, Claudio LM, Fernanda MF, Juliana LR, Abelardo SJ, et al (2012) Tripping over emerging pathogens around the world: A phylogeographical approach for determining the epidemiology of Porcine circovirus-2 (PCV-2), considering global trading. Virus Res. 163: 320–327. 10.1016/j.virusres.2011.10.019 22056846

[2] Huang YL, Pang FV, Deng MC, Chang CY, Teng CR (2014) Porcine circovirus type 2 decreases the infection and replication of attenuated classical swine fever virus in porcine alveolar macrophages. Res Vet Sci. 96: 187–195. 10.1016/j.rvsc.2013.11.020 24370262

[3] Carino R, Chardi AS, Segales J (2010) Subcellular Immunolocalization of Porcine Circovirus Type 2 (PCV2) in Lymph Nodes from Pigs with Post-weaning Multisystemic Wasting Syndrome (PMWS). J Comp Pathol (142(4)) 291–299. 10.1016/j.jcpa.2009.12.001 20096850

[4] Pejsak Z, Podgórska K, Truszczyński M, Karbowiak P, Stadejek T (2010) Efficency of different protocols of vaccination against porcine circovirus type 2 (PCV2) in a farm affected by postweaning multisystemic wasting syndrome (PMWS). Comp Immunol Microbiol Infect Dis. 33: e1–e5. 10.1016/j.cimid.2009.09.006 19910048

[5] Ge XN, Wang F, Guo X, Yang HC (2012) Porcine circovirus type 2 and its associated diseases in China. Virus Res. 164: 100–106. 10.1016/j.virusres.2011.10.005 22023739

[6] McClenahan SD, Krause PR, Uhlenhaut C (2011) Molecular and infectivity studies of porcine circovirus in vaccines. Vaccine. 29: 4745–4753. 10.1016/j.vaccine.2011.04.087 21569811

[7] Grau-Roma L, Fraile L, Segalés J (2011) Recent advances in the epidemiology, diagnosis and control of diseases caused by porcine circovirus type 2. Vet J. 187: 23–32. 10.1016/j.tvjl.2010.01.018 20211570

[8] Segales J (2012) Porcine circovirus type 2 (PCV2) infections: Clinical signs, pathology and laboratory diagnosis. Virus Res. 164: 10–19. 10.1016/j.virusres.2011.10.007 22056845

[9] Fossum C, Hjertner B, Lövgren T, Fuxler L, Charerntantanakul W, et al (2014) PCV2 on the spot-A new method for the detection of single porcine circovirus type 2 secreting cells. J. Virol. Methods 196: 185–192. 10.1016/j.jviromet.2013.10.042 24269204

[10] Huang LP, Lu YH, Wei YW, Guo LJ, Liu CM (2011) Development of a blocking ELISA for detection of serum neutralizing antibodies against porcine circovirus type 2. J. Virol. Methods 171: 26–33. 10.1016/j.jviromet.2010.09.023 20923690

[11] Huang LP, Lu YH, Wei YW, Guo LJ, Wu HL, et al (2011) Construction and biological characterisation of recombinant porcine circovirus type 2 expressing the V5 epitope tag. Virus Res. 161: 115–123. 10.1016/j.virusres.2011.05.015 21641944

[12] Che YL, Zhuang XS, Shi YT, Wang LB, Wei H, et al (2011) Establishment of indirect ELISA method to detect Porcine Circovirus 2 antibody. Chinese Agricultural Science Bulletin. 27: 290–294.

[13] Zhang WT, Wei F, Wang JL, Xiao YQ, Sheng ZQ (2012) Establishment of immunochromatographic method for rapid detection of antibody of porcine circovirus type 2. Chinese Journal of Preventive Veterinary Medicine. 34: 728–731.

[14] Chang GN, Hwang JF, Chen JT, Tsen HY, Wang JJ (2010) Fast Diagnosis and Quantification for Porcine Circovirus Type 2 (PCV-2) Using Real-Time Polymerase Chain Reaction. J Microbiol Immunol Infect. 43: 85–92. 10.1016/S1684-1182(10)60014-X 20457423

[15] Vilcek S, Vlasakova M, Jackova A (2010) LUX real-time PCR assay for the detection of porcine circovirus type. J. Virol. Methods 165 216–221. 10.1016/j.jviromet.2010.01.023 20138916

[16] Zheng LL, Wang YB, Li MF, Chen HY, Guo XP, et al (2013) Simultaneous detection of porcine parvovirus and porcine circovirus type 2 by duplex real-time PCR and amplicon melting curve analysis using SYBR Green. J. Virol. Methods 187: 15–19. 10.1016/j.jviromet.2012.06.024 22771739

[17] Perez LJ, Perera CL, Frías MT, Núñez JI, Ganges L, et al (2012) A multiple SYBR Green I-based real-time PCR system for the simultaneous detection of porcine circovirus type 2, porcine parvovirus, pseudorabies virus and Torque teno sus virus 1 and 2 in pigs. J. Virol. Methods 179: 233–241. 10.1016/j.jviromet.2011.11.009 22119629

[18] Vlasakova M, Jackova A, Leskova V, Vilcek S (2012) Development of a Plexor real-time PCR assay for the detection of porcine circovirus type 2. J. Virol. Methods 179: 311–315. 10.1016/j.jviromet.2011.11.014 22155430

[19] Chen HT, Zhang J, Sun DH, Chu YF, Cai XP, et al (2008) Rapid detection of porcine circovirus type 2 by loop-mediated isothermal amplification. J. Virol. Methods. 149: 264–268. 10.1016/j.jviromet.2008.01.023 PMC7112855 18355932

[20] Park C, Kim BS, Kim YH, Cho HS (2011) Development of serodiagnostic surface plasmon resonance imaging assay for the detection of antibodies to porcine circovirus type 2. Korean J Vet Serv. 34(1): 1–4.

[21] Hu JD, Hu JF, Luo FK, Li W, Jiang GL, et al (2009) Design and validation of a low cost surface plasmon resonance bioanalyzer using microprocessors and a touch-screen monitor. Biosens. Bioelectron 24: 1974–1978. 10.1016/j.bios.2008.10.033 19112014

[22] Hu J D, Li W, Wang TT, Lin ZL, Jiang M, et al (2012) Development of a label-free and innovative approach based on surface plasmon resonance biosensor for on-site detection of infectious bursal disease virus (IBDV). Biosens. Bioelectron 31: 475–479. 10.1016/j.bios.2011.11.019 22138467

